# Human Papillomavirus-16 E7 Interacts with Glutathione S-Transferase P1 and Enhances Its Role in Cell Survival

**DOI:** 10.1371/journal.pone.0007254

**Published:** 2009-10-13

**Authors:** Anna M. Mileo, Claudia Abbruzzese, Stefano Mattarocci, Emanuele Bellacchio, Paola Pisano, Antonio Federico, Vittoria Maresca, Mauro Picardo, Alessandra Giorgi, Bruno Maras, M. Eugenia Schininà, Marco G. Paggi

**Affiliations:** 1 Department of Development of Therapeutic Programs, CRS, Regina Elena Cancer Institute, Rome, Italy; 2 IRCCS Casa Sollievo della Sofferenza - Mendel Institute, Rome, Italy; 3 San Gallicano Dermatological Institute, Rome, Italy; 4 Department of Biochemical Sciences, Sapienza University of Rome, Rome, Italy; University of Hong Kong, Hong Kong

## Abstract

**Background:**

Human Papillomavirus (HPV)-16 is a paradigm for “high-risk” HPVs, the causative agents of virtually all cervical carcinomas. HPV E6 and E7 viral genes are usually expressed in these tumors, suggesting key roles for their gene products, the E6 and E7 oncoproteins, in inducing malignant transformation.

**Methodology/Principal Findings:**

By protein-protein interaction analysis, using mass spectrometry, we identified glutathione S-transferase P1-1 (GSTP1) as a novel cellular partner of the HPV-16 E7 oncoprotein. Following mapping of the region in the HPV-16 E7 sequence that is involved in the interaction, we generated a three-dimensional molecular model of the complex between HPV-16 E7 and GSTP1, and used this to engineer a mutant molecule of HPV-16 E7 with strongly reduced affinity for GSTP1.When expressed in HaCaT human keratinocytes, HPV-16 E7 modified the equilibrium between the oxidized and reduced forms of GSTP1, thereby inhibiting JNK phosphorylation and its ability to induce apoptosis. Using GSTP1-deficient MCF-7 cancer cells and siRNA interference targeting GSTP1 in HaCaT keratinocytes expressing either wild-type or mutant HPV-16 E7, we uncovered a pivotal role for GSTP1 in the pro-survival program elicited by its binding with HPV-16 E7.

**Conclusions/Significance:**

This study provides further evidence of the transforming abilities of this oncoprotein, setting the groundwork for devising unique molecular tools that can both interfere with the interaction between HPV-16 E7 and GSTP1 and minimize the survival of HPV-16 E7-expressing cancer cells.

## Introduction

Human Papillomaviruses (HPVs) are small DNA viruses with a marked propensity for infecting epithelial tissues. The numerous HPV genotypes are subdivided into “low-risk” HPVs, which cause benign neo-formations, and “high-risk” HPVs, which cause lesions with a predisposition to carcinogenic transformation. High-risk HPVs are associated with ano-genital carcinomas (principally cancers of the uterine cervix) [1], [2], oropharyngeal squamous cell carcinomas [3], and cutaneous malignancies (non-melanoma skin cancers) [4]. In particular, HPV-16 and HPV-18 are the causative agents of at least 90% of cervical cancers and are linked to more than 50% of other ano-genital cancers. In a vast majority of these tumors, which are usually diagnosed several years after HPV infection, a physical integration of the viral genome into cancer cell chromosomes is observed. Such integration often partially disrupts the HPV genome, but the E6 and E7 viral genes are usually retained and constitutively expressed by the host cell. This suggests key roles for their respective gene products, the E6 and E7 oncoproteins, in inducing malignant transformation [2], [5], [6]. These viral proteins act as multifaceted devices that re-program host cell functions at multiple levels, weakening the normally tight links between cellular differentiation and proliferation. In spite of being only 98 amino acids in length, the E7 protein of HPV-16 contains many features that have evolved to assist viral replication in the host cell. The expression of these oncoproteins favors the rise of and selection for clones with a high frequency of transformation. The most well understood and probably crucial role of HPV-16 E7 is its ability to neutralize the function of the RB family of tumor and growth suppressor proteins. In addition, several other HPV-16 E7 functions that can act synergistically to favor HPV replication in host cells have been described [7]–[9].

The central role of HPV-16 E7 in human oncogenesis prompted us to search for novel cellular targets of this viral oncoprotein. We screened for the host cell proteins that interact with recombinant HPV-16 E7 (UniProtKB/Swiss-Prot accession number P03129) by fishing with a tagged HPV-16 E7 construct, with subsequent identification by mass spectrometry. Using this approach, we identified glutathione S-transferase P1-1 (GSTP1; UniProtKB/Swiss-Prot accession number P09211) as the preeminent cellular partner of HPV-16 E7. Glutathione S-transferases (GST) are a family of homodimeric enzymes that play a pivotal role in cell detoxification by catalyzing the conjugation of many endogenous and exogenous hydrophobic electrophiles (such as xenobiotics) with reduced glutathione, thus neutralizing their effects within the cellular environment [10]. Remarkably, GSTP1 also possesses a regulatory function: in form of reduced monomer, it interacts with the C-terminus of c-Jun N-terminal kinase (JNK) and negatively regulates the ability of JNK to phosphorylate the Jun protein. In this way, JNK-mediated signal transduction, which can lead to apoptosis, is down-regulated [11], [12]. For these reasons, GSTP1 appears to be crucial for cell survival and inhibition of apoptosis, and is considered to be important for the survival of transformed clones and for cancer drug resistance [13]. Indeed, GSTP1 expression levels and/or polymorphisms increase in parallel with neoplastic progression and are negative prognostic factors in several human tumors [14]–[16].

In the present study, we show that HPV-16 E7 expression in immortalized human keratinocytes alters the cellular redox equilibrium and induces cell survival by stabilizing a subset of the GSTP1 protein in its reduced monomeric form.

## Results

### HPV-16 E7 physically interacts with GSTP1

**Figure 1:**
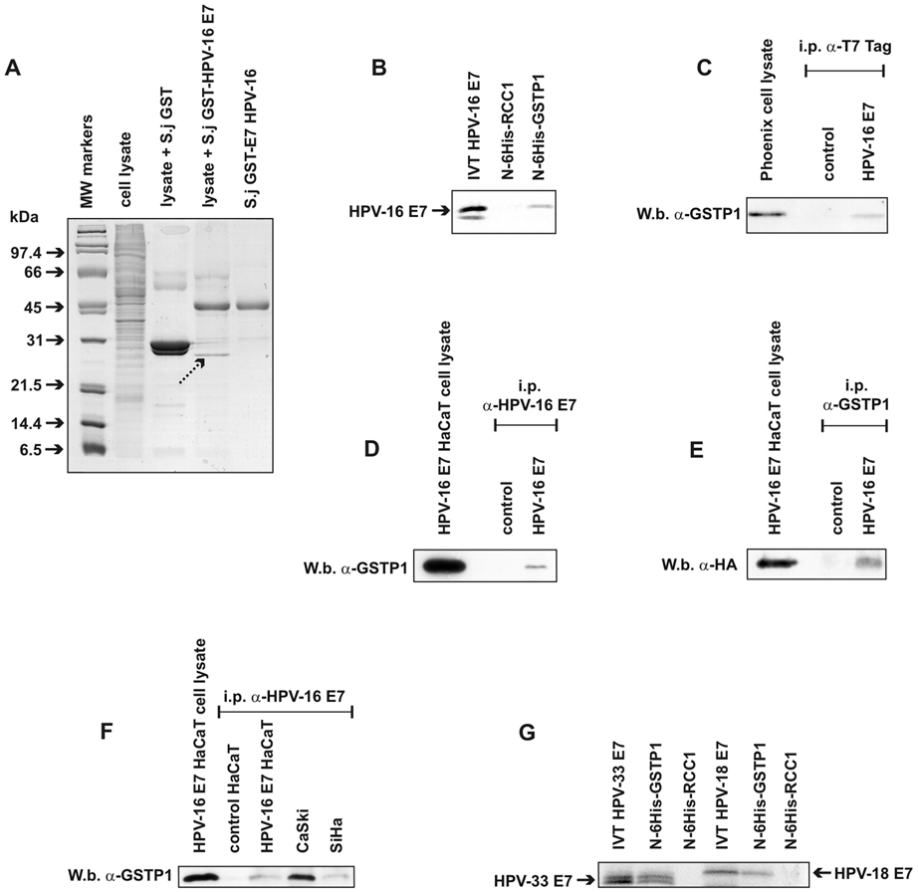
HPV-16 E7 physically interacts with GSTP1. A: HaCaT cell lysate was incubated with the *S. japonicum* GST-HPV-16 E7 chimeric protein. Co-precipitated proteins were separated by SDS-PAGE and visualized after silver staining. The dotted arrow indicates the band that was cut out and identified by peptide mass fingerprinting as human GSTP1. B: *In vitro* interaction of radiolabeled IVT HPV-16 E7 with N-6His-GSTP1 recombinant protein and lack of interaction with control N-6His-RCC1 recombinant protein. C: Western blot for GSTP1 after immunoprecipitation with an anti-T7 Tag antibody (to precipitate tagged HPV-16 E7), in control and HPV-16 E7-transfected Phoenix cells shows co-precipitation of GSTP1 only in HPV-16 E7-expressing cells. D: Western blot for GSTP1 after immunoprecipitation with an anti-HPV-16 E7 antibody in control and HPV-16 E7-expressing HaCaT cells shows co-precipitation of GSTP1 only in HPV-16 E7-expressing cells. E: Western blot using an anti-HA antibody (to detect tagged HPV-16 E7) after immunoprecipitation with an anti-GSTP1 antibody in control and HPV-16 E7-expressing HaCaT cells shows co-precipitation of HPV-16 E7. F: Western blot for GSTP1 after immunoprecipitation with an anti-HPV-16 E7 antibody in control and HPV-16 E7-expressing HaCaT cells as well as in the CaSki and SiHa cell lines expressing endogenous HPV-16 E7. G: *In vitro* interaction of radiolabeled IVT HPV-33 E7 and HPV-18 E7 with N-6His-GSTP1 recombinant protein and lack of interaction with control N-6His-RCC1 recombinant protein.

To identify novel cellular partners of the E7 protein from “high-risk” HPVs, recombinant E7 from HPV strain 16 was N-terminally tagged with the *S. japonicum* GST (S.j GST) and incubated in cell lysates from HaCaT immortalized human keratinocytes [17]. Proteins that were entrapped using immobilized glutathione as selective bait for the recombinant S.j GST-HPV16 E7 were resolved by SDS-PAGE. A number of specific bands were identified in samples incubated with S.j GST-HPV-16 E7 but not with S.j GST alone. Among these bands, a protein with an apparent molecular mass of approximately 23–25 kDa was prominent (Figure 1A, dotted arrow). This band was excised, proteolyzed by trypsin and identified by peptide mass fingerprinting as human glutathione S-transferase P1-1 (GSTP1). In this context, no other bands were analyzed.

Because HaCaT GSTP1 might interact *per se* with immobilized glutathione, it was necessary to validate this interaction using alternative experimental procedures. To this end, we generated a recombinant protein with a His tag added to the N-terminus (N-6His tag) of human GSTP1 which enables binding to immobilized nickel (Ni-NTA-agarose). This protein was incubated with radiolabeled *in vitro* translated (IVT) HPV-16 E7. Our result showed that recombinant GSTP1 indeed interacted specifically with IVT HPV-16 E7, whereas recombinant regulator of chromosome condensation 1 (RCC1) [18], which was used as an irrelevant 6-His fusion protein control, did not (Figure 1B).

To investigate the ability of HPV-16 E7 to interact with GSTP1 *in vivo*, we transfected the Phoenix cell line [19] with pcDNA3T7 Tag (control) or pcDNA3T7 Tag HPV-16 E7 plasmids. After immunoprecipitation using an anti-T7 antibody, Western blot analysis detected co-precipitated GSTP1 only in HPV-16 E7-expressing cells (Figure 1C).

Subsequently, we expressed HPV-16 E7 in HaCaT keratinocytes via the pLXSN retroviral vector (see Materials and Methods). HA-tagged HPV-16 E7 protein expression was confirmed by Western blotting. In parallel, a cell clone bearing the empty retroviral vector (control) was also produced (Figure S1). After immunoprecipitation using an anti-HPV-16 E7 antibody, Western blot analysis revealed the co-precipitation of GSTP1 only in HPV-16 E7-expressing cells (Figure 1D). Alternatively, in the same cells, following immunoprecipitation using an anti-GSTP1 antibody, the presence of co-precipitated HPV-16 E7 was detectable by Western blot (using an anti-HA antibody) only in HPV-16 E7-expressing cells (Figure 1E). In addition, using an anti-HPV-16 E7 antibody we immunoprecipitated HPV-16 E7 in the CaSki [20] and SiHa [21] cervical carcinoma cell lines, known to express HPV-16 E7 endogenously [22], [23], GSTP1 also co-precipitated with the viral oncoprotein in these cells, as detected by Western blot analysis (Figure 1F).

In order to assess whether GSTP1 was able to interact with E7 oncoproteins from “high-risk” HPVs other than HPV-16, we challenged recombinant N-6His-GSTP1 with radiolabeled IVT HPV-33 and HPV-18 E7 molecules, which interacted *in vitro* specifically with recombinant GSTP1, but not with recombinant RCC1, which was used as a control (Figure 1G).

### Identification of the amino acid sequences mediating the interaction between HPV-16 E7 and GSTP1

**Figure 2:**
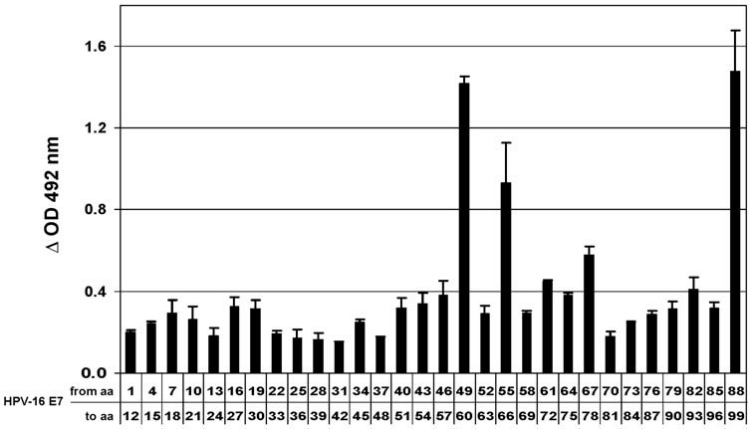
Identification of HPV-16 E7 peptide sequences involved in the interaction with GSTP1. The entire HPV-16 E7 amino acid sequence, reproduced as 12-mers with a 9 amino acid overlap, was synthesized on 30 different polypropylene rods. The histogram represents the ability of each 12-mer oligopeptide to bind recombinant GSTP1. The histogram corresponding to the 30^th^ rod refers to amino acids 88–99 because a Gly has been added to the last C-terminal HPV-16 E7 amino acid in order to complete the 12-mer. Values are averages of three different experiments±SD.

To identify the regions of the HPV-16 E7 molecule involved in its interaction with GSTP1, we took advantage of the PepSets™ technique (see Materials and Methods). A set of oligopeptides was synthesized on polypropylene rods as 12-mers with a 9 amino acid overlap, to cover the entire length of HPV-16 E7 (98 amino acids). Rod-bound peptides were then assayed for their ability to bind to recombinant GSTP1 with immunoenzymatic measurements of the amount of GSTP1 captured by each rod. The results of these experiments identified the central (aa 49–60) and C-terminal portions (aa 89–98) of HPV-16 E7 as the most important regions for its binding to GSTP1 (Figure 2). Interestingly, these two regions fall within the CR3 zinc finger domain.

### Docking of HPV-16 E7 to GSTP1

**Figure 3:**
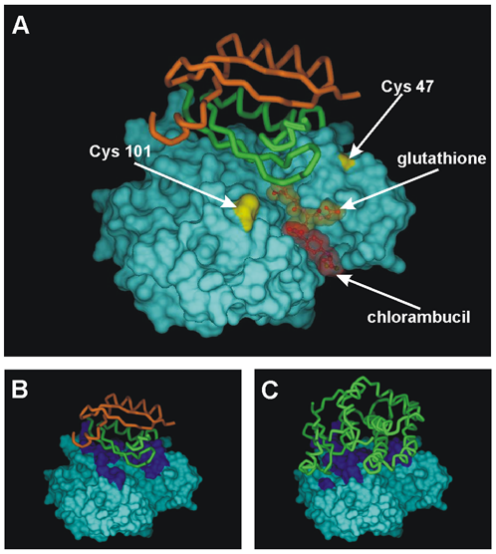
Docking of HPV-16 E7 to GSTP1. A: Docking of the GSTP1 monomer (from chain A of PDB entry 1AQW, represented as a protein surface) and the HPV-16 E7 CR3 dimer (modeled in this study and represented as a protein backbone with the two subunits distinguished by green and orange colors). The portions of the GSTP1 surface that contribute the Cys 47 and Cys 101 residues are highlighted in yellow. In the drawing, we have retained the structure of one glutathione molecule co-crystallized with GSTP1 in the PDB entry 1AQW to show its binding position (enzyme G-site). To show the enzymatic region that binds substrates (H-site of GSTP1), we have included the structure of the anticancer drug chlorambucil reproducing the same binding position as in its co-crystal with GSTP1 (PDB structure 21GS). B: In the docking model, the region of the GSTP1 monomer (surface representation) that is in contact with the HPV-16 E7 CR3 dimer (orange and green tube representation) is depicted in dark blue. C: The region of contact between one GSTP1 subunit (surface representation) and the other GSTP1 subunit (green tube representation) in the enzyme homodimer (PDB entry 1AQW) is depicted in dark blue. In panels B and C, regions of contact on the GSTP1 surfaces are identified as residues located within 5 Å of the ligand (HPV-16 E7 CR3 or the second GSTP1 unit) and appear to partially overlap.

**Figure 4:**
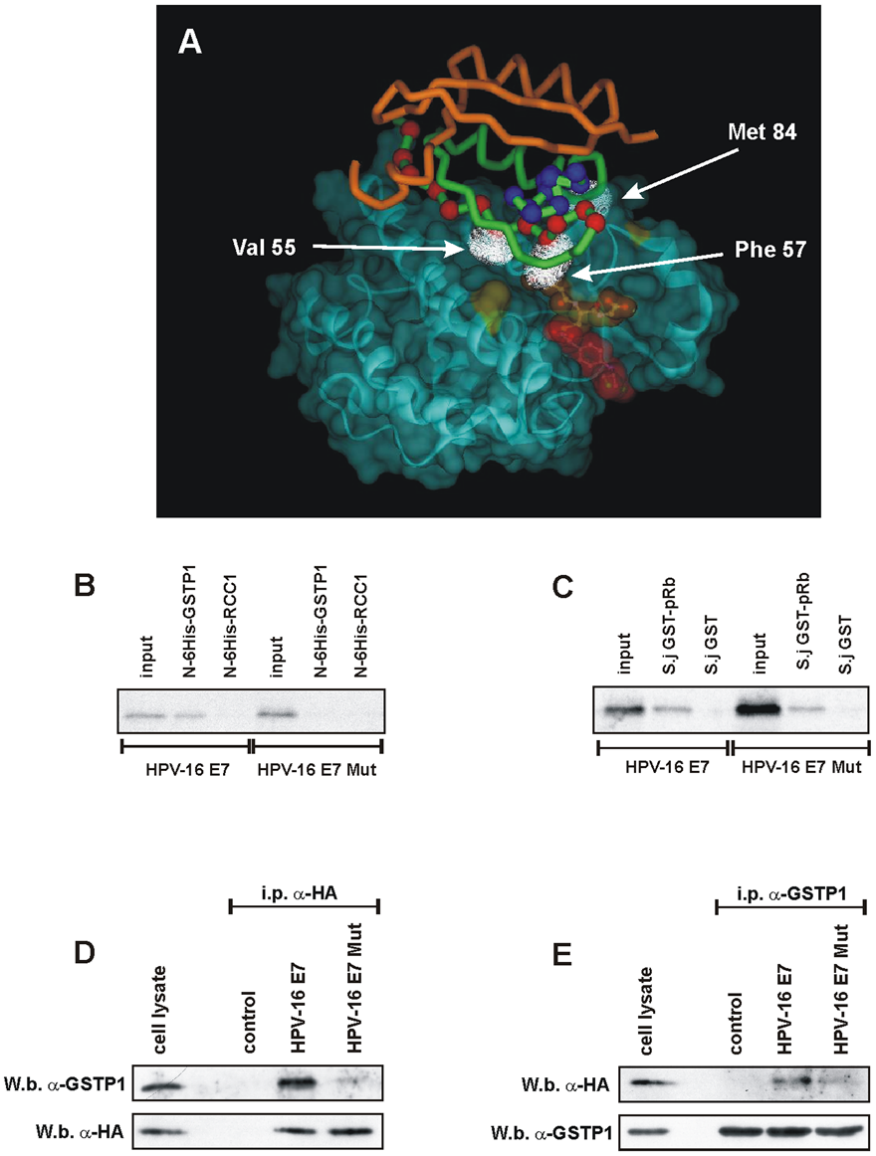
Generation of HPV-16 E7 Mut and its *in vitro* interaction with GSTP1 and pRb. A. Legend as in Figure 3A. The red spheres shown on one HPV-16 E7 subunit (green tube representation) highlight the alpha-carbon atoms of amino acid residues 49–60, while the blue spheres highlight the alpha-carbons of amino acid residues 88–98 (see Figure 2). The residues Val 55, Phe 57 and Met 84, mutated in HPV-16 E7 Mut, are highlighted by the white clouds. B: *In vitro* interaction of radiolabeled IVT HPV-16 E7, but not of HPV-16 E7 Mut, with N-6His-GSTP1 recombinant protein. N-6His-RCC1 recombinant protein was used as a negative control. C: *In vitro* interaction of radiolabeled IVT HPV-16 E7 and HPV-16 E7 Mut with recombinant *S. japonicum* GST-pRb protein. *S. japonicum* GST was used as a negative control. D: Western blot for GSTP1 after immunoprecipitation with an anti-HA antibody (to precipitate tagged HPV-16 E7 and HPV-16 E7 Mut), in control, HPV-16 E7- and HPV-16 E7 Mut-expressing HaCaT cells shows a less efficient co-precipitation of GSTP1 in HPV-16 E7 Mut-expressing cells; the same membrane was probed using an anti-HA antibody to assess the efficiency of the immunoprecipitation procedure. Cell lysate was from HPV-16 E7-infected HaCaT cells. E: Reverse co-precipitation: Western blot for HA (to detect tagged HPV-16 E7 and HPV-16 E7 Mut) after immunoprecipitation with an anti-GSTP1 antibody in control, HPV-16 E7- and HPV-16 E7 Mut-expressing HaCaT cells shows a less efficient co-precipitation of HPV-16 E7 Mut with GSTP1 when compared to HPV-16 E7; the same membrane was probed using an anti-GSTP1 antibody to assess the efficiency of the immunoprecipitation procedure. Cell lysate was from HPV-16 E7-infected HaCaT cells.

Docking of the modeled CR3 domain of HPV-16 E7 onto a GSTP1 monomer was accomplished with the Hex program, including calculations of both shape complementarity and electrostatic contributions. The best HPV-16 E7 CR3/GSTP1 interaction model is shown in Figure 3A. Interestingly, the strongly interacting peptide 49–60 (see Figure 2) corresponds to a portion of HPV-16 E7 that dwells nicely in a groove in the GSTP1 protein in our modeled complex (see below Figure 4A). This intermolecular interaction does not appear to be mediated by disulfide formation involving cysteines from the oligopeptide and GSTP1. The docking also suggests that the interaction with HPV-16 E7 involves the same GSTP1 surface region that participates in homodimerization with a second GSTP1 molecule, as seen in the native enzyme homodimer in the Protein Data Bank (PDB) structure 1AQW (Figures 3B and 3C). It was also observed that HPV-16 E7 binding does not impose any apparent steric hindrance on the residues forming the active site of the enzyme, the glutathione binding site (G-site, occupied by a glutathione molecule in Figure 3A), or the substrate binding site (H-site, occupied by chlorambucil in Figure 3A). In fact, the addition of excess reduced glutathione (GSH) (up to a 10-fold molar ratio between GSH and GSTP1) did not modify the amount of GSTP1 bound to the HPV-16 E7-derived oligopeptides (data not shown). Furthermore, GSTP1 interacts directly with only one of the two subunits of the HPV-16 E7 dimer, leaving the other subunit free to potentially interact with another GSTP1 monomer.

### Generation of a HPV-16 E7 mutant with impaired ability to bind to GSTP1

Based on the docking model shown in Figure 3, we engineered the HPV-16 E7 sequence to obtain a mutant protein carrying alanine substitutions on residues predicted to be located at the interface of the interaction between E7 and GSTP1. In particular, the mutant, named HPV-16 E7 Mut, was substituted at Val 55, Phe 57, and Met 84 (Figure 4A), based on the following considerations: 1) these three amino acids are poorly conserved in E7 proteins across the various Papillomavirus types; 2) and according to our CR3 domain model, they do not appear to contribute to the stabilization of the HPV-16 E7 dimer; and 3) their side chains form the largest hydrophobic and solvent-exposed region on the surface of the CR3 dimer, suggesting that this group of residues might contribute at least in part to the interaction between HPV-16 E7 and other proteins. Interestingly, we also noticed that three hydrophobic amino acids are also present in other of the most aggressive HPV strains (18, 31, 33, 45 and 59) at positions corresponding to the residues Val 55, Phe 57 and Met 84 of HPV-16 E7, while the hydrophobic character of the same amino acid sites is poorly conserved across all HPV strains. Figure 4A shows how the two strong interacting peptides found by means of the PepSets™ technique are positioned in the structure of the docked HPV-16 E7-GSTP1 complex. Although, in the model, the interaction of HPV-16 E7 with GSTP1 appears to involve the 49–60 12-mer predominantly, with little contribution from the C-terminal 88–98 peptide, the comparably strong interaction observed for the corresponding peptides with the PepSets™ technique could be attributed to conformational differences. In particular, the interaction of the C-terminal peptide with GSTP1 might be enhanced when this peptide is in the form of a structurally less-restrained 12-mer, as opposed to when it is embedded into the folded CR3 domain.

Radiolabeled IVT HPV-16 E7 and HPV-16 E7 Mut were incubated with recombinant GSTP1. Subsequent SDS-PAGE and autoradiography experiments showed that, *in vitro*, using recombinant proteins, only the wild-type HPV-16 E7 molecule interacted with GSTP1; recombinant RCC1 was used as a negative control (Figure 4B). In addition, the molecular perturbations induced in HPV-16 E7 Mut did not interfere with the ability of the oncoprotein to bind *in vitro* to the oncosuppressor protein pRb, a molecular target of E7 (Figure 4C).

HaCaT cells were subsequently engineered to express the HPV-16 E7 Mut molecule (Figure S1), in order to evaluate its effects in various biological assays when compared to the wild-type molecule. Either wild-type or mutant HPV-16 E7 was expressed as HA-tagged proteins. To assay the ability of HPV-16 E7 Mut to interact with GSTP1 *in vivo*, control, HPV-16 E7-infected and HPV-16 E7 Mut-infected HaCaT cells were lysed and immunoprecipitation using an anti-HA antibody was performed. Subsequently, the amount of co-precipitated GSTP1 was determined by Western blotting. The efficiency of the immunoprecipitation procedure was assessed by probing the same membrane with an anti-HA antibody (Figure 4D). Reverse co-immunoprecipitation was also carried out, by immunoprecipitating the same lysates with an anti-GSTP1 antibody and determining by means of Western blotting the amount of HPV-16 E7 and HPV-16 E7 Mut coprecipitated, using an anti-HA antibody. The efficiency of the immunoprecipitation procedure was assessed by probing the same membrane with an anti-GSTP1-antibody (Figure 4E). Both *in vivo* experiments confirmed the interaction between HPV-16 E7 and GSTP1, and also showed that the ability of HPV-16 E7 Mut to interact with GSTP1 was drastically reduced, but not abolished.

### GSTP1 activities in HPV-16 E7-expressing and HPV-16 E7 Mut-expressing HaCaT cells

To explore the *in vivo* role of HPV-16 E7 in epithelial cells, we investigated the effects of HPV-16 E7 and HPV-16 E7 Mut expression on GSTP1 total protein content and assayed its known functions in our HaCaT model system.

**Figure 5:**
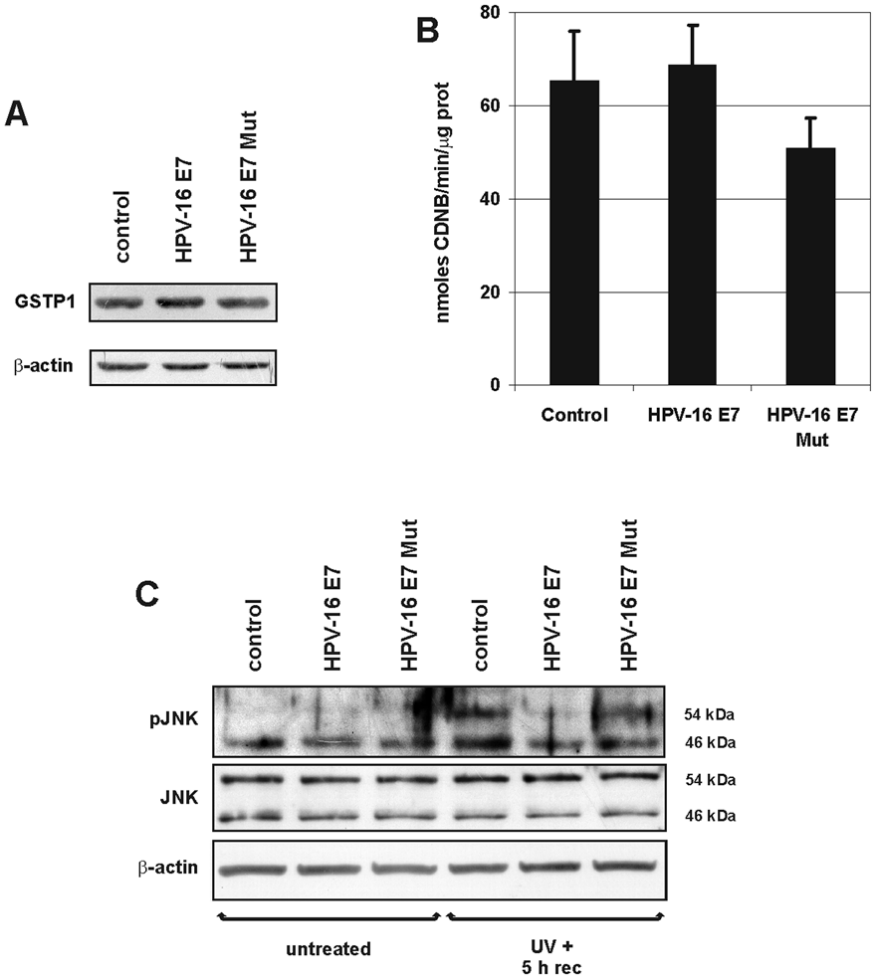
GSTP1 activities in HPV-16 E7- and HPV-16 E7 Mut-expressing HaCaT cells. A: A representative Western blot for GSTP1 in control cells and HPV-16 E7- and HPV-16 E7 Mut-expressing HaCaT cells. Blots were normalized against β-actin levels. B: GSTP1 enzymatic activity in control, in HPV-16 E7- and HPV-16 E7 Mut-expressing HaCaT cells (see Materials and Methods). Values are averages of three different experiments±SEM and their variations were not statistically significant. C: JNK protein levels in control and in HPV-16 E7- and in HPV-16 E7 Mut-expressing HaCaT cells that were untreated or cells that were sampled after induction of oxidative stress by exposure to UV (UVB, 5 mJ/cm^2^ for 1 min) with a 5 h recovery period, evaluated using an antibody specific for the phosphorylated form of JNK (pJNK) and another antibody that detects total JNK levels (JNK, performed on a separate twin gel). Only after UV exposure did HPV-16 E7-expressing cells display a markedly reduced JNK phosphorylation, while a lower reduction in JNK phosphorylation was detectable in HPV-16 E7 Mut-expressing cells.

Western blot analysis for GSTP1 showed comparable amounts of protein in control cells and HPV-16 E7- and HPV-16 E7 Mut-expressing HaCaT cells (Figure 5A). RNA extracted from these cells was assayed for GSTP1 transcript levels by real-time quantitative PCR, showing only insignificant differences among the cells (data not shown). GSTP1 catalytic activity was not significantly affected in HPV-16 E7 and HPV-16 E7 Mut-expressing cells, with respect to the control samples (Figure 5B), suggesting that enzymatic GSTP1 activity was not altered by the binding with its viral oncoprotein.

Besides its enzymatic activity, the pro-survival properties of GSTP1 via the JNK cascade should be considered. In fact, GSTP1, in form of reduced monomer, enhances cell survival by inhibiting JNK phosphorylation and its signaling cascade activity; phosphorylated (activated) JNK can phosphorylate Jun, a member of the AP1 transcriptional regulator, thus eliciting a JNK-dependent apoptotic cascade [11], [12]. Therefore, we assayed JNK phosphorylation levels in control cells and in HPV-16 E7- or HPV-16 E7 Mut-expressing HaCaT cells, untreated or after UV irradiation. In non-irradiated cells (untreated), no evident changes in pJNK expression were found; on the other hand, after UV irradiation, cells expressing HPV-16 E7 displayed markedly reduced JNK phosphorylation when compared to the related control, while the reduction was less evident in cells expressing HPV-16 E7 Mut. In contrast, total intracellular JNK levels appeared essentially unaffected (Figure 5C).

### HPV-16 E7 influence on the balance between oxidized and reduced GSTP1

**Figure 6:**
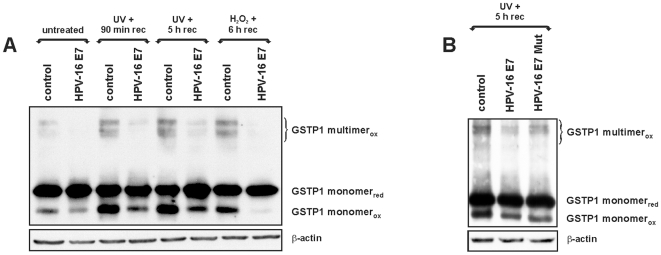
HPV-16 E7 influences the balance between oxidized and reduced GSTP1. A: Western blots for GSTP1 after non-reducing SDS-PAGE in control and in HPV-16 E7-infected HaCaT cells under normal conditions (untreated) or after induction of oxidative stress by exposure to UV (UVB, 5 mJ/cm^2^ for 1 min) or hydrogen peroxide (0.5 mM H_2_O_2_ for 30 min). In the first case, detection was performed after a 90 min or 5 h recovery period, while in the second case detection was performed after a 6 h period. In all cases, HPV-16 E7 expression was accompanied by a drastic decrease in the GSTP1 oxidized multimeric form (GSTP1 multimer_ox_) and of the lowest band, which represents the oxidized GSTP1 monomer (GSTP1 monomer_ox_). GSTP1 monomer_red_ denotes the reduced form. B: Western blots for GSTP1 after non-reducing SDS-PAGE in control, HPV-16 E7- and HPV-16 E7 Mut-infected HaCaT cells after exposure to UV (UVB, 5 mJ/cm^2^ for 1 min) and 5 h recovery. The mutant oncoprotein appeared less efficient in protecting GSTP1 from oxidation. In both panels, blots were normalized against β-actin levels that were determined in matching gels run under reducing conditions.

The GST proteins are known to undergo polymerization via interchain disulfide bridges in an oxidizing environment To assay whether or not this behavior could be modulated by HPV-16 E7, control and HPV-16 E7-infected HaCaT cells were assayed by Western blot for GSTP1 protein levels after non-reducing SDS-PAGE [24], [25]. Under basal (untreated) conditions, this assay revealed a decrease in the amount of multimeric oxidized GSTP1 in HPV-16 E7-expressing cells compared to that in control cells. This effect was even more evident after the induction of oxidative stress by UV irradiation or exposure to H_2_O_2_, which caused a substantial increase in the oxidized forms of the enzyme in control cells. In all cases, HPV-16 E7-expressing cells displayed a marked decrease not only in the multimeric oxidized form, but also in the monomeric oxidized form of GSTP1 (Figure 6A). This monomeric oxidized form is generated by an intrasubunit disulfide bond between the Cys-47 and Cys-101 residues [24], [25] and is distinguishable from the reduced form because of its higher electrophoretic mobility. By assaying the effect of HPV-16 E7 Mut expression on the equilibria between multimeric and monomeric, and oxidized or reduced GSTP1 in UV-irradiated HaCaT cells, we found that the mutant oncoprotein appeared less proficient than the wild-type in protecting GSTP1 from oxidation (Figure 6B). This result could be interpreted in light of the partially impaired binding of the mutant oncoprotein to the GSTP1 molecule. The residual binding ability could be also attributable to the C-terminal region of the HPV-16 E7 (see Figure 2).

### HPV-16 E7 expression and redox equilibrium

We assayed a set of biochemical parameters to estimate the possible interference from either wild-type or mutant HPV-16 E7 with the cellular redox equilibrium. We determined in control and HPV-16 E7- and HPV-16 E7 Mut-expressing HaCaT cells: a) the amount of thiobarbituric acid reactive substances (TBARS), which are the degradation end-products of peroxidated lipids; b) the content of polyunsaturated fatty acids (PUFAs), which are the first targets of lipoperoxidative insult; c) the enzyme activity levels of superoxide dismutase (SOD), catalase, and glutathione peroxidase (GSH-Px); and d) the amount of GSH, which is the most important free radical scavenger. The results, shown in Table 1, indicate significant differences between control and HPV-16 E7-expressing cells, with respect to parameters related to lipid peroxidation (TBARS and PUFA), cytosolic redox equilibrium (SOD, catalase, and GSH-Px) and GSH content, all of which are consistent with the hypothesis that HPV-16 E7 induces oxidative stress. In cells expressing HPV-16 E7 Mut, significant differences with the control were found in the SOD, catalase, and GSH-Px activities, as well as in the total GSH content, while significant differences with HPV-16 E7-expressing cells were only observed in regards to TBARS and GSH-Px. In summary, expression of either HPV-16 E7 or HPV-16 E7 Mut appears to be linked to the condition of marked cellular stress.

**Table 1:** Modifications induced by HPV-16 E7 and HPV-16 E7 Mut expression on the redox equilibrium of HaCaT cells.

	Parameter	Control	HPV-16 E7	HPV-16 E7 Mut
**Lipid peroxidation**	TBARS (nmol/mg prot.)	0.37±0.01	0.71±0.04**	0.42±0.09**^◊◊^**
	PUFA (%)	25.36±0.41	22.92±0.91*	23.76±0.83
**Cytosol redox equilibrium**	SOD (U/mg prot.)	31.91±2.05	38.29±2.01*	37.92±1.09*
	Catalase (U/mg prot.)	6.33±0.85	7.45±1.01*	8.52±0.93*
	GSH-Px (mU/mg prot.)	0.56±0.03	0.85±0.03**	0.74±0.02**/**^◊^**
	GSH (nmol/mg prot.)	193.53±2.74	235.67±19.85*	240.00±18.73*

Results are the averages±SD of three different determinations performed in duplicate. In the HPV-16 E7 column, asterisks indicate statistical significance between the values reported for control and for HPV-16 E7-expressing cells (* = *P*<0.05; ** = *P*<0.01). In the HPV-16 E7 Mut column, asterisks indicate statistical significance between the values reported for control and for HPV-16 E7 Mut-expressing cells (* = *P*<0.05; ** = *P*<0.01), while diamonds indicate statistical significance between the values reported for HPV-16 E7 and HPV-16 E7 Mut-expressing cells (**^◊^** = *P*<0.05; **^◊◊^** = *P*<0.01).

### Role of GSTP1 and HPV-16 E7 in cell survival after UV exposure

To assay the role of GSTP1 and HPV-16 E7 in cell survival after UV exposure, we used two cellular models: 1) the HaCaT cells and 2) the MCF-7 human breast carcinoma cell line [26]. The latter lacks GSTP1 expression [27] because of promoter hypermethylation [28]. As for HaCaT keratinocytes, we expressed HPV-16 E7 in MCF-7 cells via the pLXSN vector. A control clone was produced in parallel. HPV-16 E7 expression in both cell lines was confirmed by Western blotting. As expected, GSTP1 expression was undetectable in both control and HPV-16 E7-expressing MCF-7 cells (Figure S2A).

**Figure 7:**
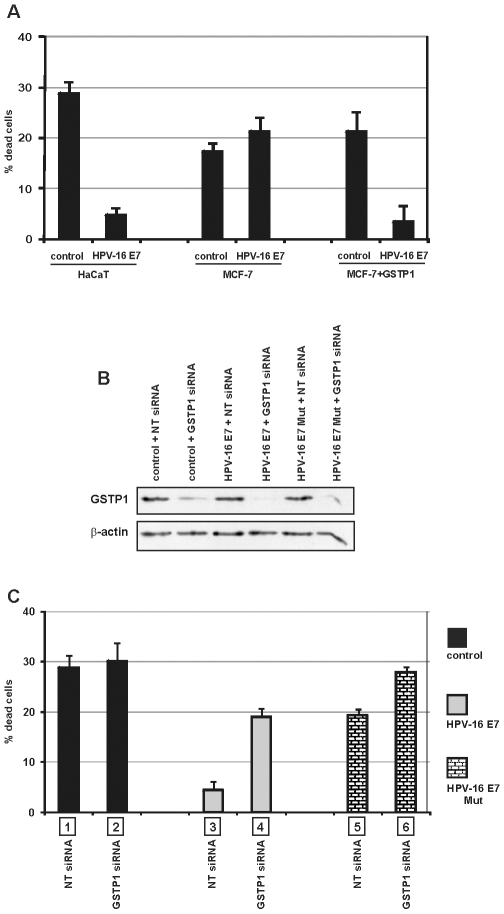
Roles of HPV-16 E7 and GSTP1 and their interaction in cell survival after UV exposure. A: After exposure to UV radiation (UVB, 5 mJ/cm^2^ for 1 min), control and HPV-16 E7-expressing HaCaT cells showed a significant correlation between HPV-16 E7 expression and cell survival (*p* = 0.000003, ***). Under the same conditions, GSTP1-deficient MCF-7 cells displayed no significant differences in survival, and thus no HPV-16 E7-related protection. Forced expression of GSTP1 in control and HPV-16 E7-infected MCF-7 cells restored the ability of HPV-16 E7 to protect against UV-induced cell death (*p* = 0.0046, **). Values are the averages of three independent experiments performed in triplicate±SEM. B: Control, HPV-16 E7- and HPV-16 E7 Mut-infected HaCaT cells were transfected with non-targeting (NT) siRNA or with siRNA targeting GSTP1. After 72 h, GSTP1 siRNA-treated cells showed decreased GSTP1 protein expression. C: Cells, exposed to UV radiation as above, were collected and the number of Trypan blue-negative (live) cells was determined. The differential survival rate between cells unexposed and exposed to UV radiation was then calculated, and the results were expressed as percentages of dead cells after UV exposure. The results show a remarkable effect of HPV-16 E7 on cell survival (bars 1 vs. 3), a less effective protection by HPV-16 E7 Mut (bars 1 vs. 5), and the consequences of GSTP1 silencing in HPV-16 E7- (bars 3 vs. 4) and HPV-16 E7 Mut- expressing cells (bars 5 vs. 6). This panel indicates the role of GSTP1 in HPV-16 E7-induced cell survival and the importance of the binding ability of the oncoprotein in inducing the GSTP1-mediated increase in survival. Values are the average of three independent experiments performed in triplicate±SEM. Statistical significance of the reported values: bars 1 vs. 3 *p* = 0.00004, ***; bars 1 vs. 5 *p* = 0.0009, ***; bars 3 vs. 5 *p* = 0.000004, ***; bars 3 vs. 4 *p* = 0.0002, ***; bars 5 vs. 6 *p* = 0.0001, ***; bars 2 vs. 4 *p* = 0.0288, *; bars 4 vs. 6 *p* = 0.0007, ***.

To estimate the specific role of GSTP1, we forced its expression in control and HPV-16 E7-infected MCF-7 cells by electroporation (MCF-7+GSTP1 cells) (Figure S2B). Aliquots of control and HPV-16 E7-expressing cells were exposed to UV radiation. After five hours, the cells were collected and the number of Trypan blue-negative (live) cells was counted. The differential survival rate between cells that were unexposed and exposed to UV radiation was determined and expressed as the percentage of dead cells after UV exposure. The protective effect of the viral oncoprotein was evident and statistically significant for the HaCaT cells, as described [29], [30], and for GSTP1-expressing MCF-7 cells, but not for the GSTP1-deficient MCF-7 cells (Figure 7A).

To define the synergy between GSTP1 and HPV-16 E7 and the role of their interaction in enhancing cell survival after UV exposure, we used siRNA to down-regulate GSTP1 protein levels. Control, HPV-16 E7- and HPV-16 E7 Mut-expressing HaCaT cells were transfected with either control non-targeting (NT) siRNA or siRNA targeting GSTP1. After 72 h, aliquots of the cells were analyzed for GSTP1 expression in order to assess the efficiency of siRNA-mediated silencing, which ranged from 75% to 90%. The results of a representative experiment are shown in the Western blot in Figure 7B. Cells were then aliquoted, exposed to UV and processed as described above. In NT siRNA-transfected HaCaT cells, HPV-16 E7 expression significantly increased cell survival (Figure 7C, bars 1 and 3), confirming the pro-survival role of the viral oncoprotein. However, this effect was reduced considerably in GSTP1-silenced cells (Figure 7C, bars 2 and 4). In this setting, HPV-16 E7 Mut expression was only able to partially protect HaCaT cells from death (Figure 7B, bars 1 and 5), and this effect was drastically reduced in GSTP1-silenced cells (Figure 7C, bars 5 and 6).

## Discussion

In HaCaT cells expressing either wild-type or mutant HPV-16 E7, it is possible to trace oxidative events, which are highlighted by an increase in lipid peroxidation. However, the increase of GSH content and of SOD, catalase, and GSH-Px activities demonstrates an active response from these cells to oxidative injuries. These results are confirmed by those from other studies in HPV-16 E7-transfected HaCaT cells [31] or HPV-16-infected keratinocytes, where viral oncoproteins expression is associated with increased resistance to UV radiation [32]. GSTP1 total protein level and its catalytic activity in HaCaT cells were not influenced by either the expression of HPV-16 E7 (a result in accord with other laboratories) [33], or the expression of HPV-16 E7 Mut, a mutant form of the oncoprotein engineered to reduce its binding to GSTP1. In agreement with previous results [29], the changes induced by expression of the viral oncoprotein appear associated with enhanced HaCaT cell survival after UV exposure.

In this scenario, the direct link observed between the HPV-16 E7 viral factor and the GSTP1 protein confers a pivotal role to this physical interaction in enacting the survival capabilities modulated by GSTP1. GSTP1 acts as an intrinsic negative regulator of apoptosis essentially through its inhibition of JNK signaling, and we envision a mechanism by which GSTP1 is activated via physical interaction with HPV-16 E7. Indeed, we have shown that HPV-16 E7 strongly decreases the levels of oxidized GSTP1, thus increasing the level of the reduced form, which interferes with JNK signaling [11]. In addition, the concomitant up-regulation of GSH levels in HPV-16 E7-infected cells might act synergistically to increase the concentration of reduced GSTP1.

Multimerization of GSTP1 through intermolecular disulfide formation, which occurs under oxidizing conditions, is associated with enzyme inactivation. Cys 47 is the residue with the highest reactivity towards thiol-specific reagents, and its covalent modification is accompanied by a loss of GST activity [34]. GSTP1 is also inactivated when Cys 47 forms an intramolecular disulfide bond with Cys 101 [24]. On the basis of these observations, and as suggested by our model in which HPV-16 E7 occupies the space between Cys 47 and Cys 101 of GSTP1 and prevents both intramolecular and intermolecular disulfide formation, we propose that HPV-16 E7 binding might protect GSTP1 against inactivation via oxidative attacks at Cys 47 and/or Cys 101. According to our model, the binding of HPV-16 E7 to GSTP1 and GSTP1 homodimerization should be mutually exclusive, because these two different complexes involve mostly overlapping regions of the enzyme surface. Although we lack experimental evidence that allows us to state whether GSTP1 retains GST activity when bound by HPV-16 E7, it is remarkable how closely the conformation of this complex emulates the pattern of intermolecular contacts in the GSTP1 homodimer. We hypothesize that the role of HPV-16 E7 is to establish a subset of GSTP1 molecules that is inaccessible to oxidative attack, thus creating a reservoir of reduced monomeric GSTP1. This hypothesis is strongly supported by the results obtained from the expression in the HaCaT model system of a HPV-16 E7 mutant that binds GSTP1 less efficiently. Indeed, the mutant molecule we engineered, HPV-16 E7 Mut, while unable to bind 6-His-GSTP1 *in vitro* as a recombinant protein, displayed a certain residual effectiveness *in vivo* in binding native GSTP1, in safeguarding it from oxidation and in protecting the cells from UV-induced cell death. It is noteworthy that the peculiar perturbation we generated in the HPV-16 E7 mutant molecule by amino acid substitutions in the CR3 region did not appreciably affect binding with pRb, which interacts with HPV-16 E7 mostly via the CR2 region, though involvement of the C-terminal region has been reported [9], [35].

The results we obtained in the GSTP1-deficient MCF-7 cell line further support the active role of GSTP1 in HPV-16 E7-related survival, where HPV-16 E7 expression did not protect MCF-7 cells from UV radiation-induced damage, unless GSTP1 was forcibly co-expressed in this model system. The central role of GSTP1 in mediating the pro-survival effects of HPV-16 E7 expression in HaCaT cells is also clearly outlined by the results obtained from silencing GSTP1 expression via siRNA, while the role of the physical interaction between these two factors is indicated by the results generated from the expression of the mutant form of the oncoprotein. Indeed, the survival of HPV-16 E7-expressing HaCaT cells after UV irradiation was significantly decreased when GSTP1 protein levels were reduced by RNAi. Notably, in this model system, the survival of control HaCaT cells after UV exposure did not seem to rely on GSTP1 in the absence of HPV-16 E7 expression. In addition, the results obtained in cells expressing HPV-16 E7 Mut clearly highlighted the substantial role of the physical interaction between the oncoprotein and GSTP1 in modulating its anti-apoptotic, JNK-mediated activity.

These data underscore a clear role for HPV-16 E7 in enhancing cell survival and provide additional evidence for the transforming capabilities of the E7 oncoprotein from high-risk HPVs. Indeed, HPV-16 E7 expression can a) accelerate or induce entry into the cell cycle [8], [9], b) induce mitotic abnormalities hence increasing genomic instability [36], [37], and c) increase cell survival, at least via the pro-survival mechanisms discussed above. All of these characteristics might act in concert to facilitate the rise and selection of transformed cell clones and the progression of cancer [38].

A closer examination of the molecular changes elicited by HPV viral oncoproteins via their physical interaction with cellular components will certainly help to elucidate the network of effects generated by these viruses during infection and transformation. These findings will help in the development of unique molecular tools that can prevent and treat virus-induced tumors.

## Materials and Methods

### Ethics Statement

Due to the fact that neither humans nor other animals have been used in this research, an Ethics Statement is not required for this work.

### Plasmids, constructs and primers

Full-length HPV-16 E7 coding sequences were amplified by PCR from HPV-16 cDNA template and cloned into the pcDNA3T7 Tag plasmid and pLXSN retroviral vector in frame with the N-terminal hemagglutinin (HA) epitope tag. Sequences of oligonucleotide primers were as follows:

***HPV-16 E7 forward oligonucleotide (FW)*** **.** 5′GATCGAATTCATGGCTTACCCATACGATGTTCCAGATTACGCTCATGGAGATACACCTACATTGCATG-3′

***HPV-16 E7 reverse oligonucleotide (RV)*** **.**

5′-GATCGGATCCGCAGGATCAGCCATGGTAGA-3′

ORF encoding human GSTP1 was synthesized by RT-PCR from HaCaT cells RNA using “First strand cDNA synthesis kit” (Amersham Biosciences, Piscataway, NJ) with the following specific primers:

***GSTP1 FW*** **.**

5′GGGG**ACAAGTTTGTACAAAAAAGCAAGGCT**TACCGCCCTACACCGTGGTCTATTTC-3′

***GSTP1 RV*** **.**

5′GGGG**ACCACTTTGTACAAGAAAGCTGGGT**CTCACTGTTTCCCGTTGCCATTGATGGG-3′

To facilitate GSTP1 cloning into the Gateway Entry vector pDONR201 (Invitrogen, Carlsbad, CA), AttB1 and AttB2 tails (indicated in boldface) were added to the 5′ end of both FW and RV primers.

Recombinant HPV-16 E7 and human pRb were expressed in BL21 *E. coli* cells as *S. japonicum* GST fusions (pGEX-4T-1 and pGEX-2T, respectively; Amersham Biosciences, Piscataway, NJ), whereas human GSTP1 was expressed in BL21 *E. coli* cells as an N-terminally 6His-tagged protein (pDEST17; Gateway cloning system, Invitrogen). Proteins were purified by glutathione-Sepharose 4B (Amersham Biosciences) (*S. japonicum* GST-tagged) or on Ni-affinity (Qiagen, Milan, Italy) (N-6His-tagged) column chromatography according to manufacturer's instructions.

### Recovery of specific bands and protein identification

Bands representing HaCaT cellular proteins specifically interacting with GST-HPV-16 E7 were excised from the gel, incubated for 20 min in the dark with destaining solutions provided in the SilverQuest^TM^ Silver Staining kit (Invitrogen), and subjected to tryptic proteolysis, using the In-gel Digest kit (Millipore Corporation, Billerica, MA) as described [39]. The mass spectrometry analyses were performed with a MALDI-ToF Voyager-DE STR instrument (Applied Biosystems, Framingham, MA), equipped with a 337 nm nitrogen laser and operating in reflector mode. All mass spectra were internally calibrated with two tryptic autolytic peptides (*m/z* 842.5100 and 2807.3145). The mass lists obtained from each PMF analyzed, after exclusion of the contaminant mass values (autolytic tryptic peptides and tryptic human keratin fragments) by the PeakErazor program (http://www.protein.sdu.dk/gpmaw/Help/PeakErazor/peakerazor.html), were used to search for protein candidates in (human) NCBLnr database consulting the Mascot search engine (http://www.matrixscience.com). All searches respected the following parameters: one missed cleavage, 50 ppm measurement tolerance, oxidation at methionine (as variable modification). Positive identifications were accepted when, with at least five peptide masses matched, *P* values (the probability that the observed match is a random event) were lower than 0.05.

### Site-directed mutagenesis

Mutations were introduced into the HPV-16 E7 plasmid construct using the QuickChange Site-Directed Mutagenesis kit (Stratagene, La Jolla, CA) according to the manufacturer's instructions. To obtain triple point mutation, the following mutagenesis primer pairs were used to perform two consecutive rounds of PCR amplification cycles in the HPV-16 E7 cDNA construct plasmid:

***Double mutation (V_55_A – F_57_A)*** **.** FW: 5′-CCCATTACAATATTGCAACCGCTTGTTGCAAGTGTGACTCTACGC-3′

RV: 5′-GCGTAGAGTCACACTTGCAACAAGCGGTTGCAATATTGTAATGGG-3′

***Single mutation (M_84_A)*** **.** FW: 5′-CCGTACGTTGGAAGACCTGTTAGCAGGCACACTAGGAATTGTGTG-3′

RV: 5′-CACACAATTCCTAGTGTGCCTGCTAACAGGTCTTCCAACGTACGG-3′

For all primers, mutagenized positions are underlined.

PCR amplifications were performed under the following conditions: denaturation at 95°C for 2 min, followed by 12 cycles of denaturation at 95°C for 30 s, annealing at 55°C for 50 s and extension at 68°C for 5 min.

Destruction of the methylated, parental DNA plasmid by *DpnI* digestion followed by *E.coli* cells transformation allowed the introduction of the desired mutations. The mutation site and the fidelity of HPV-16 E7 coding sequence were confirmed by DNA sequencing.

### Cell lines

The cell lines used were cultured in DMEM (Invitrogen) plus 10% fetal bovine serum (Invitrogen) at 37°C in a 5% CO_2_ atmosphere. HaCaT cells were cultured at low density (max. 40% confluent), and CaSki cells using DMEM+GlutaMAX-I (Invitrogen).

### Incubation of recombinant HPV-16 E7 with cell lysate and SDS-PAGE analysis

HaCaT cells were lysed in 50 mM Tris-Cl, pH 7.4 with 5 mM EDTA, 250 mM NaCl, and 0.1% Triton X-100 (lysis buffer) plus protease inhibitors (1 mM PMSF, 10 µg/ml aprotinin and leupeptin) for 30–40 min on ice. Chimeric GST-HPV-16 E7 protein (1.5 µg) was added to the lysate and incubated for 100 min at 4°C. Recombinant proteins, and any bound molecules were collected by adding insoluble glutathione-Sepharose 4B (Amersham Biosciences). Precipitated complexes were analyzed by 10% SDS-PAGE, followed by silver staining (Bio-Rad Laboratories, Hercules, CA).

### PepSets™ technology

To precisely identify the amino acids involved in the interaction between HPV-16 E7 and GSTP1, we employed PepSets™ technology (Mimotopes Pty Ltd, Clayton, Victoria, Australia), as described [30], [40], [41]. The reactivity of these rod-bound peptides was then assayed for their ability to physically interact with recombinant GSTP1. The amount of protein captured by each rod was detected via a modified immunoenzyme assay using an anti-GSTP1 polyclonal antibody (Oxis Research, Portland, OR.) at a 1∶2,000 dilution. At the end of the procedure, plates were analyzed in a microtiter reader.

### Modeling of the docking of HPV-16 E7 to GSTP1

The interaction between HPV-16 E7 and GSTP1 has been assumed to involve the dimeric and monomeric forms of the two proteins, respectively. The dimer of the CR3 domain of HPV-16 E7 in the amino acid interval 48 to 98 was modeled with the program MODELLER© release 8v2 [42] using the structure of the dimer of the homologous CR3 domain of HPV-1A E7 (PDB code 2B9D) as a template. The GSTP1 monomer was represented by chain A of the PDB structure 1AQW, with the assumption that it does not undergo conformational changes in the absence of crystallographically bound molecules (the second bound monomer in the GSTP1 homodimer, glutathione and salts). Docking of the HPV-16 E7 dimer with the GSTP1 monomer was performed using the program Hex v4.5 (Department of Computing Science, University of Aberdeen, Aberdeen AB24 3UE, Scotland, UK; http://www.csd.abdn.ac.uk/hex/), allowing full translational and rotational searching and recording the 500 best score solutions.

### In vitro binding assay

For the experiment shown in Figure 1B, *in vitro* translation of HPV-16 E7 was performed as described [18], using ^35^S-methionine as a radioactive label. For the experiments shown in Figure 1G, *in vitro* translation of HPV-33 E7 and of HPV-18 E7, and in Figure 4, *in vitro* translation of HPV-16 E7 and HPV-16 E7 Mut, assays were performed using ^35^S-cysteine as a radioactive label. Aliquots of reaction mixtures containing *in vitro*-translated proteins were added to Ni-NTA agarose beads coupled to 4 µg of recombinant N-6His RCC1 (negative control) or recombinant N-6His GSTP1. Alternatively, aliquots of reaction mixtures containing the *in vitro*-translated proteins were added to glutathione-Sepharose 4B coupled to 1 µg of *S. japonicum* GST-pRb or *S. japonicum* GST (negative control). Incubation, 12% SDS-PAGE, and detection were performed as described [18].

### GST activity

GST catalytic activity was assayed using 1-chloro-2,4-dinitrobenzene (CDNB) as an electrophilic substrate (Sigma) in the presence or absence of 4 µM ethacrynic acid (EA), an inhibitor of GSTP1 activity [43], to quantify the contribution of GSTP1 activity to overall cellular GST enzyme activity. Conjugation of the thiol group of glutathione to the CDNB substrate was monitored by recording changes in absorbance at 340 nm between 0.5 and 6 min after initiating the reaction at 22°C. GST catalytic activity was expressed as nmol of CDNB/min/µg of protein. Results are averages±SEM of three different determinations performed in duplicate.

### Transfections and infections

Calcium phosphate transfection of Phoenix cells with pcDNA3T7 Tag and pcDNA3T7 Tag HPV-16 E7 plasmids were performed using standard procedures. Transfection of pDEST26 or pDEST26-GSTP1 in control- and HPV-16 E7-infected MCF-7 cells was done by electroporation using the Gene Pulser II apparatus (Bio-Rad) according to Manufacturer's instructions.

For the infection procedure, Phoenix amphotropic packaging cells were transfected with HA-tagged pLXSN (Clontech), pLXSN-HPV-16-E7 or pLXSN-HPV-16-E7 Mut as described [30]. HaCaT or MCF-7 infection by Phoenix cell supernatants and clone selection were carried out as described [30].

### In vivo co-immunoprecipitations

Phoenix or HaCaT cells were lysed in lysis buffer (50 mM Tris-Cl, pH 7.4, 5 mM EDTA, 250 mM NaCl, 50 mM NaF, 0.1% Triton X-100, 0.1 mM Na_3_VO_4_, 1 mM PMSF, 10 µg/ml Leupeptin) for 30 min on ice. Lysates were centrifuged at 14,000×g for 10 min at 4°C. Lysates were cleared and incubated with one of the following antibodies: anti-T7 Tag, monoclonal, (Novagen, Madison, WI, 1∶100 dilution); anti-HPV-16 E7, monoclonal, (ed-17, Santa Cruz Biotechnology, Santa Cruz, CA, 1 µl∶100 µg lysate); anti-GSTP1, polyclonal (Assay Designs, Ann Arbor, MI.; 1∶100 v:v dilution) for 2 h, followed by incubation with protein A-Sepharose (for anti-T7 Tag and anti-HPV-16 E7 primary Abs) or G-Sepharose (for anti-GSTP1 primary antibody) beads in lysis buffer. The beads were subjected to four rounds of washing with 1 ml of lysis buffer, then immunoprecipitates were eluted by adding electrophoresis sample buffer. Proteins were resolved through SDS-PAGE, transferred to PVDF membranes and probed with the specific antibodies indicated in the Western blotting section.

### Western blot analysis

SDS-PAGE and Western blot analysis were performed as described [18]. Where indicated, proteins were separated under non-reducing conditions in SDS-PAGE by excluding β-mercaptoethanol from the sample buffer. Membranes were probed with primary antibodies followed by horseradish peroxidase-conjugated anti-rabbit or anti-mouse immunoglobulins. Immunoreactive bands were detected using ECL reagents (Amersham Biosciences). The following antibodies and dilutions were used: anti-SAPK/JNK pAb (Cell Signaling, Beverly, MA, 1∶1,000 dilution) against total JNK; anti-phospho-SAPK/JNK (Thr183/Tyr 185) pAb (Cell Signaling, 1∶1,000 dilution) against phosphorylated forms of JNK; anti-HA-tag mAb (Cell Signaling, 1∶1,000 dilution) against influence hemagglutinin epitope; anti-GSTP1-1 pAb (Assay Designs, Ann Arbor, MI.; 1∶10,000 dilution); anti-β actin mAb (MP Biomedicals, Irvine, CA., 1∶10,000 dilution).

### Analysis of the cellular redox equilibrium

#### Thiobarbituric acid reactive substances (TBARS) analysis

Evaluation of TBARS was performed as described [44]. Standard curves were obtained using malondialdehyde (MDA) standard solutions at different concentrations (1.25, 0.5, 0.25 and 0.1 µM). TBARS values were expressed as nmol/mg of protein.

#### Poly-unsaturated fatty acids (PUFAs) analysis

Evaluation of PUFAs in the phospholipid fraction of cell membranes was performed as described [45]. Results were expressed as percent variations.

#### Antioxidant enzymatic activities

Antioxidant enzymatic activities were determined in the supernatants of native cell lysates obtained after repeated freezing and thawing in liquid nitrogen and subsequent centrifugation at 10,000 g for 10 min at 4°C. The activities of superoxide dismutase (SOD) [46], catalase [47] and glutathione peroxidase (GSH-Px) [48] were determined spectrophotometrically as described and expressed as U/mg of protein for SOD and catalase and as mU/mg of protein for GSH-Px.

#### GSH determination

GSH cellular content was estimated as described [49]. Results were expressed as nmol/mg of protein.

### siRNA transfection

For gene silencing experiments, HaCaT cells were plated at a density of 1×10^5^ cells in 35-mm dishes 24 h before transfection. For these experiments, cell density (∼30% confluence at the time of transfection) was critical for transfection efficiency.

On-Target siRNA (*SMARTpool* L-011179, Dharmacon, Inc. Lafayette, CO) or On-Target plus *siControl* (Non-targeting pool, D-001810-10 Dharmacon) were transfected (50 nM for 5 h) using Lipofectamine 2000 (Invitrogen) (10 µl per dish) according to the manufacturer's instructions. siCONTROL TOX Non Targeting siRNA (D-001500-01 Dharmacon) was used as an indicator of transfection efficiency. GSTP1 protein levels were assessed by Western blot 72 h post-transfection.

### Cell viability assays

Viability was measured by the ability of cells to exclude Trypan blue dye (Invitrogen). Trypsinized cells were incubated with 0.4% Trypan blue and examined microscopically. The percentage of cells that excluded Trypan blue was determined by counting 100 cells in triplicate per experimental condition in a hemocytometer. The differential survival rate between cells unexposed and exposed to UV radiation was determined and expressed as percentage of dead cells after UV exposure.

### Statistical analysis

Statistical analysis of the results was performed using the two-tailed Student's *t* test, with GraphPad Prism v5.01 for Windows (GraphPad Software, San Diego CA).

## Supporting Information

Materials and Methods S1 (0.03 MB DOC)

Figure S1 HPV-16 E7 and HPV-16 E7 Mut protein expression in HaCaT cells. Western blot detection of the wild-type and mutant viral oncoproteins (anti-HA Ab) in HPV-16 E7- and in HPV-16 E7 Mut-infected HaCaT cells; control cells resulted negative. The blot was normalized against β-actin levels.
(0.29 MB TIF)

Figure S2 HPV-16 E7 and GSTP1 protein expression in HaCaT and in GSTP1-deficient and in GSTP1-tranfected MCF7 cells. A: Western blot for HPV-16 E7 (anti-HA Ab) and GSTP1 in control and HPV-16 E7-infected HaCaT and MCF-7 cells, showing the expression of the viral oncoprotein in both infected cell clones and GSTP1 undetectability in the MCF-7 cells. The blot was normalized against β-actin levels. B. Western blot for HPV-16 E7 (anti-HA Ab) and GSTP1 in control and HPV-16 E7-infected HaCaT and in GSTP1-transfected MCF-7 cells, showing the expression of the viral oncoprotein in both infected cell clones and of GSTP1 in the transfected MCF-7 cells. The blot was normalized against β-actin levels.
(0.69 MB TIF)
