# Supplementary material for: Human Papillomavirus-16 E7 Interacts with Glutathione S-Transferase P1 and Enhances Its Role in Cell Survival
Source: PLoS One. 2009 Oct 13;4(10):e7254. doi: 10.1371/journal.pone.0007254 (PMC2758704; doi:10.1371/journal.pone.0007254)
Supplement: Materials and Methods S1 — (0.03 MB DOC) [file pone.0007254.s001.doc]

**Supplementary Materials and Methods**

***Relative quantification of GSTP1 gene expression by real-time RT-PCR***

One-step quantitative RT-PCR analysis for GSTP1 and cyclophilin A expression was performed using the following internal primer sequences.

Primer sequences for GSTP1:

FW: 5’-catgctgctggcagatcag-3’;

RV: 5’- gacctcatggatcagcagc-3’.

Primer sequences for cyclophilin A:

FW: 5’-TGGTCAACCCCACCGTGTTC-3’;

RV: 5’-GCCATCCAACCACTCAGTC-3’.

Primer sequences were obtained from the NCBI database (Accession number [NM_000852](http://www.ncbi.nlm.nih.gov/entrez/viewer.fcgi?db=nucleotide&val=6552334) and BC000689, respectively).

Quantitative RT-PCR for cyclophilin A was carried out on each sample as an internal control for template levels. The relative expression of human GSTP1 mRNA was calculated as described in: Chen YP, Higgins JA, Gundersen-Rindal DE (2003) Quantitation of a Glyptapanteles indiensis polydnavirus gene expressed in parasitized host, Lymantria dispar, by real-time quantitative RT-PCR. J Virol Methods 114: 125-133.
