# Supplementary figures and images for: Human Papillomavirus-16 E7 Interacts with Glutathione S-Transferase P1 and Enhances Its Role in Cell Survival
Source: PLoS One. 2009 Oct 13;4(10):e7254. doi: 10.1371/journal.pone.0007254 (PMC2758704; doi:10.1371/journal.pone.0007254)

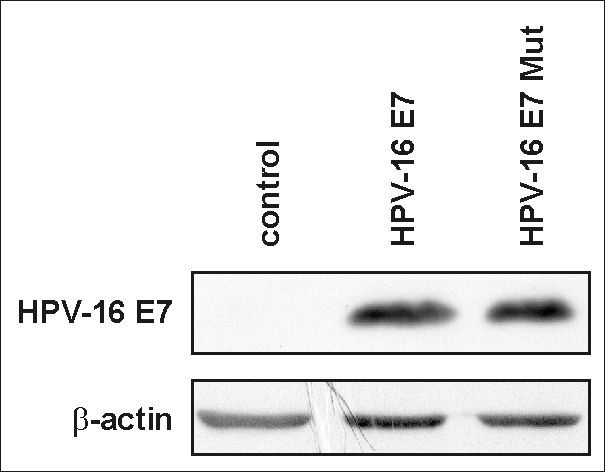

Supplement: Figure S1 — HPV-16 E7 and HPV-16 E7 Mut protein expression in HaCaT cells. Western blot detection of the wild-type and mutant viral oncoproteins (anti-HA Ab) in HPV-16 E7- and in HPV-16 E7 Mut-infected HaCaT cells; control cells resulted negative. The blot was normalized against β-actin levels. (0.29 MB TIF) [file pone.0007254.s002.tif]

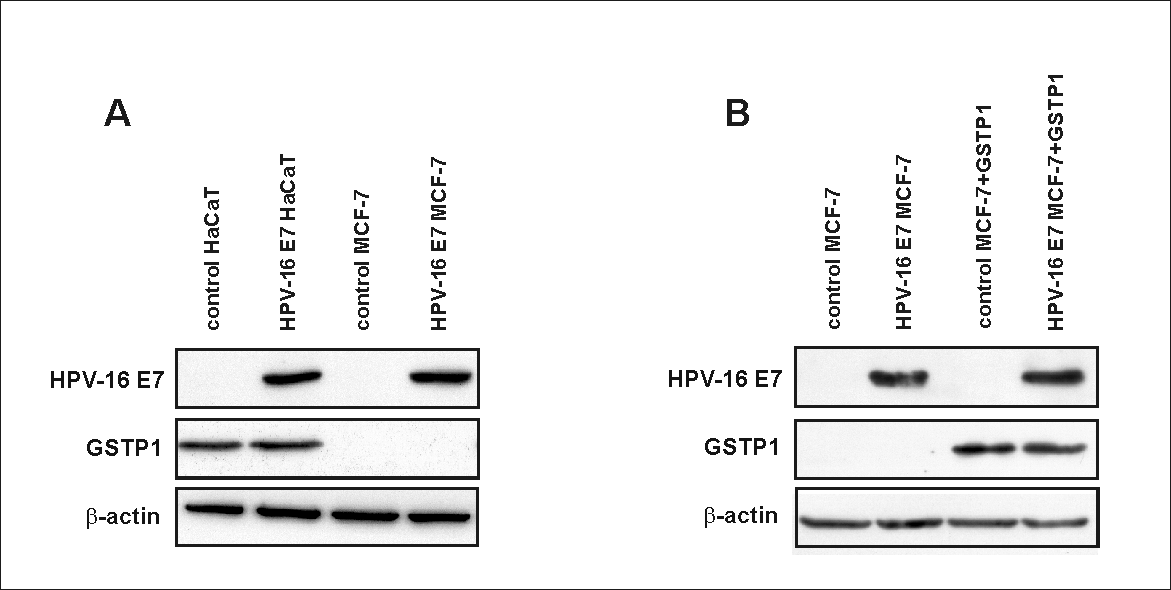

Supplement: Figure S2 — HPV-16 E7 and GSTP1 protein expression in HaCaT and in GSTP1-deficient and in GSTP1-tranfected MCF7 cells. A: Western blot for HPV-16 E7 (anti-HA Ab) and GSTP1 in control and HPV-16 E7-infected HaCaT and MCF-7 cells, showing the expression of the viral oncoprotein in both infected cell clones and GSTP1 undetectability in the MCF-7 cells. The blot was normalized against β-actin levels. B. Western blot for HPV-16 E7 (anti-HA Ab) and GSTP1 in control and HPV-16 E7-infected HaCaT and in GSTP1-transfected MCF-7 cells, showing the expression of the viral oncoprotein in both infected cell clones and of GSTP1 in the transfected MCF-7 cells. The blot was normalized against β-actin levels. (0.69 MB TIF) [file pone.0007254.s003.tif]
